# Highly Sensitive Detection of Hydrogen Peroxide in Cancer Tissue Based on 3D Reduced Graphene Oxide–MXene–Multi-Walled Carbon Nanotubes Electrode

**DOI:** 10.3390/bios14060261

**Published:** 2024-05-21

**Authors:** Shuai-Qun Yu, Pan Li, Hao-Jie Li, Ling-Jun Shang, Rui Guo, Xu-Ming Sun, Qiong-Qiong Ren

**Affiliations:** School of Medical Engineering, Xinxiang Medical University, Xinxiang 453003, China; m15225998391@163.com (S.-Q.Y.); kxx0806@163.com (P.L.); 18436264383@163.com (H.-J.L.); slj1216002129@163.com (L.-J.S.); pyguorui@sina.com (R.G.)

**Keywords:** H_2_O_2_, rGO, MXene, MWCNTs, real-time detection, cancer tissue

## Abstract

Hydrogen peroxide (H_2_O_2_) is a signaling molecule that has the capacity to control a variety of biological processes in organisms. Cancer cells release more H_2_O_2_ during abnormal tumor growth. There has been a considerable amount of interest in utilizing H_2_O_2_ as a biomarker for the diagnosis of cancer tissue. In this study, an electrochemical sensor for H_2_O_2_ was constructed based on 3D reduced graphene oxide (rGO), MXene (Ti_3_C_2_), and multi-walled carbon nanotubes (MWCNTs) composite. Three-dimensional (3D) rGO–Ti_3_C_2_–MWCNTs sensor showed good linearity for H_2_O_2_ in the ranges of 1–60 μM and 60 μM–9.77 mM at a working potential of −0.25 V, with sensitivities of 235.2 µA mM^−1^ cm^−2^ and 103.8 µA mM^−1^ cm^−2^, respectively, and a detection limit of 0.3 µM (S/N = 3). The sensor exhibited long-term stability, good repeatability, and outstanding immunity to interference. In addition, the modified electrode was employed to detect real-time H_2_O_2_ release from cancer cells and cancer tissue ex vivo.

## 1. Introduction

The death rate from cancer increases year after year, despite advances in technology [1]. The incidence of breast cancer currently accounts for 11.7% of all newly diagnosed cancer cases worldwide and has emerged as the leading cause of mortality among female cancer patients [2]. The primary diagnostic modalities for cancer include marker diagnosis, pathology diagnosis, and imaging diagnosis. The Marker Diagnostics technology is capable of detecting biomarkers produced by malignant cells in various bodily fluids, including blood, tissue fluid, secretions, and tumor tissue [3]. H_2_O_2_ is an essential signaling molecule that regulates various biological processes, including protein synthesis, immune system function, and cell differentiation and proliferation [4,5,6]. Studies demonstrate that cancer cells release more H_2_O_2_ because of higher H_2_O_2_ production or lower H_2_O_2_ scavenging capacity during abnormal tumor growth [7,8]. Therefore, measurement of H_2_O_2_ levels in cells and tissue is essential for inchoate diagnosis of cancer [9].

Traditional methods for H_2_O_2_ detection include fluorescence analysis [10], chemiluminescence [11], electrochemical analysis [12], and spectrophotometry [13]. However, these methods have certain limitations, such as expensive equipment, cumbersome operation, stringent environmental testing requirements, and susceptibility to environmental influences [14]. In contrast, electrochemical analysis methods offer the advantages of cost-effectiveness, simplicity, and high selectivity [15,16]. It has been discovered that specific nanomaterials, such as gold nanoclusters [17], platinum nanoparticles [18], graphene oxide [19], and its derivatives [20], possess the ability to mimic the activity of catalase. In addition, properties such as the excellent electrical, chemical, and structural properties of nanomaterials deserve special attention. Consequently, the utilization of nanomaterials in H_2_O_2_ sensors can effectively enhance the sensitivity and specificity of detection, attributed to their exceptional properties [21,22].

Graphene is a two-dimensional (2D) monolayer material composed of sp^2^ hybridized carbon atoms. It has garnered significant attention due to its exceptional electronic conductivity, expansive surface area, favorable biocompatibility, and impressive mechanical properties [23]. However, strong van der Waals forces between individual graphene sheets can form disordered stacks or even re-stacking, affecting its excellent electrochemical properties [24]. On the other hand, MXene is a novel two-dimensional transition metal carbide, nitride/carbonitrides [23]. The chemical formula is Mn + 1XnTx, *n* = 1, 2, 3, where M is a transition metal element, X is C or N, and Tx is a surface oxygenated functional group (e.g., –F, –OH, –O–) [25]. These oxygenated functional groups have promising applications for redox mechanisms in the field of electrochemistry [26,27]. MXene has attracted wide interest due to its excellent metal conductivity, hydrophilicity, and high specific surface area [28]. However, MXene also exhibits some limitations, such as aggregation between individual layers and weak stability [29]. These reasons lead to the underutilization of oxygen-containing functional groups, which results in poor electrochemical performance. Therefore, a mixture of MXene and graphene was employed to form a 3D porous structure through hydrothermal reduction. This approach effectively mitigates the stacking issues associated with both MXene and graphene, thereby enhancing the stability of MXene while maximizing the utilization of oxygen-containing functional groups on its surface [30]. In addition, the composites increase the electrochemical surface area and accelerate the electron transfer, which improves its electrochemical performance [31,32]. Zhao et al. developed a novel 3D Prussian blue-doped reduced graphene oxide/MXene composite aerogel (3D PB/MGA) from rGO and MXene. The 3D PB/MGA was immobilized on a glassy carbon electrode (GCE) to construct a non-enzymatic sensor for H_2_O_2_ [33].

MWCNTs possess remarkable characteristics, including a substantial specific surface area and a high electron transport capacity [34,35]. The incorporation of carbon nanotubes into graphene structures effectively mitigates the aggregation of individual graphene sheets and significantly enhances their electrochemical sensor performance [24]. After the incorporation of MWCNTs into MXene, they become entangled through π-π interactions, resulting in a rougher film surface and thereby enhancing the electroactive area of the composite [36]. Zhang et al. developed a H_2_O_2_ sensor with a wide linear range (0.05–18 mM) using MWCNTs, Ti_3_C_2_T_x_, and Pd [37].

The MCF−7 cell line is extensively employed in breast cancer research due to its well-established suitability as a model system for studying this disease [38]. The 4T1 cell line was frequently utilized to establish a murine model of breast cancer. Disease progression in the 4T1 mouse model closely mimicked clinical manifestations of advanced malignant breast cancer in humans [39]. N-formyl-L-methionyl-L-leucyl-L-phenylalanine (fMLP) is a typical representative of the chemokine N-formylated oligopeptide family. The binding of fMLP to specific receptors on the cell surface can initiate the activation of intracellular signaling pathways, resulting in cellular activation and subsequent generation of H_2_O_2_ [40]. Catalase is capable of efficiently scavenging H_2_O_2_ generated by malignant tissues.

**Scheme 1:**
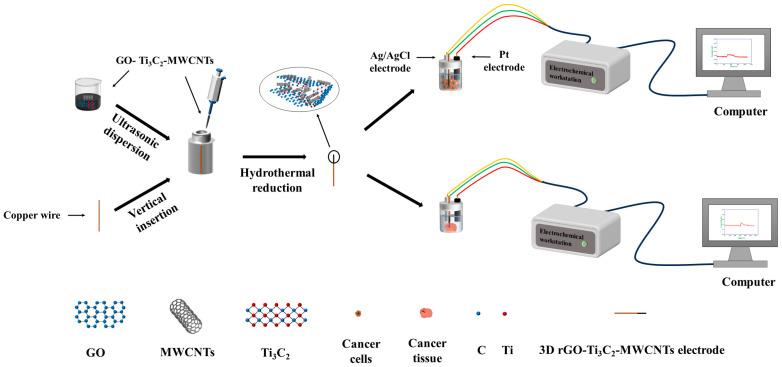
Fabrication of 3D rGO–Ti_3_C_2_–MWCNTs and the detection of H_2_O_2_ released from cancer cells and tissue.

In this study, 3D rGO immobilized MXene and MWCNTs electrode was structured for high sensitivity and selectivity detection of H_2_O_2_ (Scheme 1). The stability, interference immunity, and measurement repeatability were investigated. MCF−7 and 4T1 cells, which generate H_2_O_2_ endogenously under stimulation by fMLP, were chosen as model cells to demonstrate the electrochemical detection ability of the 3D rGO–Ti_3_C_2_–MWCNTs electrode. Finally, real-time continuous monitoring of H_2_O_2_ in mouse breast cancer tissue was achieved.

## 2. Materials and Methods

### 2.1. Chemicals and Materials

Hydrogen peroxide (H_2_O_2_, 30%), uric acid (UA, 99%), ascorbic acid (AA, 99%), dopamine (DA, 98%), sodium dihydrogen phosphate (Na_2_HPO_4_, 99%), potassium dihydrogen phosphate (KH_2_PO_4_, 99.5%), sodium chloride (NaCl, 99.99%), potassium chloride (KCl, 99.5%), dichloromethane (CH_2_Cl_2_, 99.8%), and potassium ferricyanide (K_3_[Fe(CN)_6_], 99.5%) were obtained from Macklin (Shanghai, China). All reagents were analytically pure. Graphene oxide (GO) was bought from Ashine Advanced Carbon Materials Co., Ltd. (Changzhou, China). MXene (single layer Ti_3_C_2_) was purchased from Beike 2D Materials Co., Ltd. (Beijing, China). MWCNTs (>1 μm in length, 7–15 nm in diameter) were acquired from Nanotech Port Co., Ltd. (Shenzhen, China). Cell Counting Kit−8 (CCK−8) was purchased from Boster Biotech Inc Co., Ltd. (Wuhan, China). Catalase and fMLP were obtained from Aladdin Biochemical Technology Co., Ltd. (Shanghai, China). Deionized water with a specific resistivity of 18.25 MΩ cm^−1^ was used for all experiments.

### 2.2. Preparation of the 3D rGO–Ti_3_C_2_–MWCNTs Electrode

The 3D rGO–Ti_3_C_2_–MWCNTs electrode was prepared using a simple one-step hydrothermal method. First, a mixture of GO (0.4 mg mL^−1^), Ti_3_C_2_ (0.4 mg mL^−1^), and MWCNTs (0.4 mg mL^−1^) was ultrasonically dispersed for 2 h. Second, the resulting suspension (2 mL) was injected into a 5 mL Teflon-lined autoclave suspended with copper wire (diameter 0.2 mm) and maintained at 180 °C for 4 h. After hydrothermal reduction, 3D rGO–Ti_3_C_2_–MWCNTs hydrogel film was modified on copper wire. The modified electrode was dried at room temperature. Finally, insulating wax was coated on the side of the electrode cylinder to form a disk electrode. The 3D rGO, rGO–Ti_3_C_2_, and rGO–MWCNTs electrodes were prepared using the same method.

### 2.3. Electrochemical Characterization

Electrochemical tests were carried out on an electrochemical workstation (CHI660e, Shanghai, CH Instruments Ins. Shanghai, China). All electrochemical measurements were performed using the three-electrode system. Pt electrode, Ag/AgCl electrode, and 3D rGO–Ti_3_C_2_–MWCNTs electrode were used as the counter electrode, reference electrode, and working electrode, respectively. Origin 2021 software (Origin 9.1; OriginLab, Northampton, MA, USA) was used to analyze all the measurement data. Scanning electron microscopy (SEM) (Nova 450; FEI Inc., Eindhoven, Holland) was used to investigate the morphology of the 3D rGO–Ti_3_C_2_–MWCNTs electrode. X-ray photoelectron spectroscopy (XPS, Thermo Fisher Scientific ESCALAB Xi+, Waltham, MA, USA) was used to characterize the elemental compositions of the modified electrode. The phase structure of the modified electrode was characterized via X-ray diffraction (XRD, Rigaku SmartLab, Tokyo, Japan).

### 2.4. Cell Culture and Animals

Human breast cancer cell line MCF−7 and mouse breast cancer cell line 4T1 were purchased from Servicebio Technology Co., Ltd. (Wuhan, China). Cells were cultured in Dulbecco’s modified eagle medium (DMEM) containing 10% fetal bovine serum (FBS) complemented with 100 U mL^−1^ penicillin and 100 mg mL^−1^ streptomycin and grown in a cell culture incubator with 5% CO_2_ and 37 °C. The cells were washed with 0.01 M phosphate-buffered saline (PBS) after density reached 80−90%. Then, 1mL trypsin-EDTA (0.25%) was added to dislodge cells from the bottom of the dish. The number of cells was counted by a hemocytometer. The collected cells were resuspended in a deoxygenated 0.01 M PBS solution.

The female BALB/c mice (5–6 weeks) were purchased from Vital River Laboratory Animal Technology Co., Ltd. (Beijing, China). The experimental protocol was approved by the Animal Experimentation Committee of Xinxiang Medical College and conducted in accordance with international ethical standards for the use of experimental animals. The experimental mice were kept in an animal room with suitable temperature and humidity, alternating between light and dark for 12 h. During the breeding process, we ensured that sufficient water and food were provided for experimental mice. According to animal ethics, the tumor load of experimental mice should not exceed 4000 cubic millimeters. We cultured tumors up to 500 cubic millimeters to minimize the suffering of experimental mice. We measured the tumor volume of the experimental mice twice a week. When tumor volume reached 500 cubic millimeters, the mice were euthanized. The number of experimental mice used and the suffering experienced were minimized as much as possible throughout the experiment. All mice were kept in a specific pathogen-free (SPF) grade laboratory animal house at the Stem Cell Experiment Center of Xinxiang Medical University. All experimental animals were operated in accordance with the animal ethics and guidelines of the Laboratory Animal Center of Xinxiang Medical University. In total, 1.0 × 10^6^ 4T1 cells were injected subcutaneously into the lateral thigh of BALB/c mice. The experimental mice were cultured for 2−3 weeks in a pathogen-free animal house. In about two weeks, cancer tissues that increased to 500 cubic millimeters were separated. The cancer tissues were washed three times with 0.01 M PBS solution and soaked in 5 mL of deoxygenated 0.01 M PBS solution for electrochemical detection.

### 2.5. Detection of H_2_O_2_ in Live Cells and Tissue

Amperometric detection of H_2_O_2_ release from cells and tissue was performed with the 3D rGO–Ti_3_C_2_–MWCNTs electrode. After a steady background was obtained, 50 μL fMLP (1 mg mL^−1^) was injected into the buffer. In 100 s, 50 μL catalase (500 U mL^−1^) was injected. The response currents were recorded under an applied potential of −0.25 V.

## 3. Results and Discussion

### 3.1. Physical Characterization

**Figure 1:**
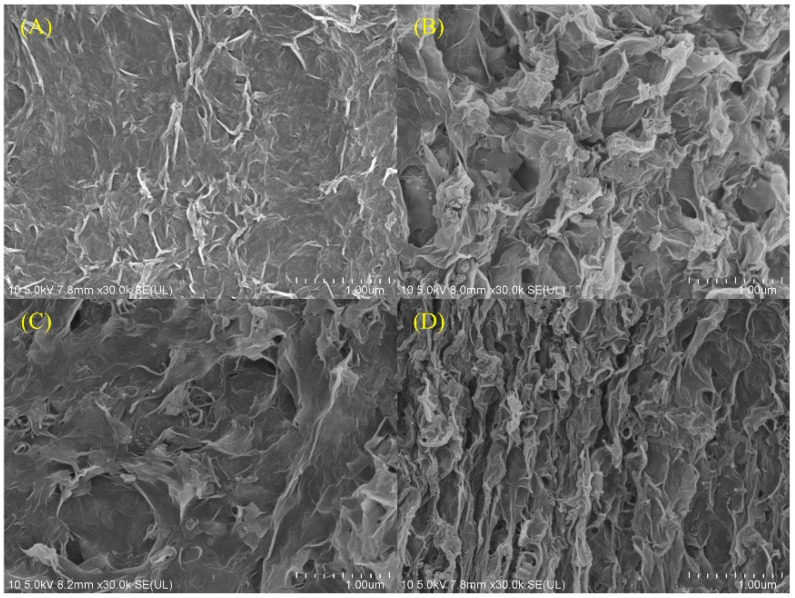
SEM images of (**A**) 3D rGO, (**B**) 3D rGO–Ti_3_C_2_, (**C**) 3D rGO–MWCNTs, and (**D**) 3D rGO–Ti_3_C_2_–MWCNTs at the scale of 1 µm.

The electrode surfaces of 3D rGO, rGO–Ti_3_C_2_, rGO–MWCNTs, and rGO–Ti_3_C_2_–MWCNTs were characterized by SEM. The rGO electrode surface exhibited a typical wrinkled surface morphology on the scale of 1 µm (Figure 1A). The formation of rGO with a nanosheet structure was achieved through hydrothermal reduction of GO [41]. rGOs can easily aggregate because of the strong van der Waals forces and π-π stacking interactions [42]. Hydrothermal reduction resulted in the formation of 3D rGO–Ti_3_C_2_ with numerous surface pores and effectively prevented rGO stacking [26,27]. The entanglement of MWCNTs on rGO nanosheets was achieved through the hydrothermal reduction method. Thus, the surface of 3D rGO–MWCNTs became rougher compared to that of 3D rGO (Figure 1C) [42]. The introduction of Ti_3_C_2_ and MWCNTs into the GO solution simultaneously resulted in an increased surface roughness and wrinkling of the 3D rGO–Ti_3_C_2_–MWCNTs electrode. The results suggested that the presence of Ti_3_C_2_ and MWCNTs promoted analyte aggregation, enhanced active sites, and improved electrocatalytic activity. In addition, XRD and XPS characterizations are presented in Figure S1.

### 3.2. Direct Electrochemical Behavior of the 3D rGO–Ti_3_C_2_–MWCNTs Electrode

**Figure 2:**
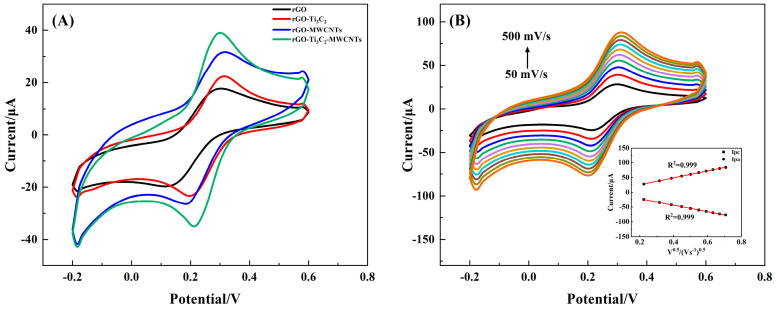
(**A**) CV of 3D rGO, rGO–Ti_3_C_2_, rGO–MWCNTs, and rGO–Ti_3_C_2_–MWCNTs electrodes in 5 mM K_3_[Fe(CN)_6_] and 0.1 M KCl at scan rates of 100 mV s^−1^. (**B**) CV of the 3D rGO–Ti_3_C_2_–MWCNTs electrode in 5 mM K_3_[Fe(CN)_6_] and 0.1 M KCl at scan rates of 50, 100, 150, 200, 250, 300, 350, 400, 450, and 500 mV s^−1^ (inset: plot of the oxidation and reduction peak currents vs. square root of the scan rate).

Cyclic voltammetry (CV) analyses were carried out in a mixture of 5 mM K_3_[Fe(CN)_6_] and 0.1 M KCl to characterize the electron transfer process. The peak-to-peak separation (ΔEp) between oxidation peak potential and reduction peak potential responds to the degree of reversibility of the electrochemical reaction. The smaller ΔEp indicated a greater reversibility degree, higher electron transfer efficiency, and more practical application value [43]. ΔEp of 3D rGO, rGO–Ti_3_C_2_, rGO–MWCNTs, and rGO–Ti_3_C_2_–MWCNTs electrodes were 192 mV, 132 mV, 140 mV, and 112 mV, respectively, at scan rates of 100 mV s^−1^. Compared to the 3D rGO electrode, 3D rGO–Ti_3_C_2_ and rGO–MWCNTs electrodes had significantly increased peak current and decreased ΔEp (Figure 2A). The 3D rGO–Ti_3_C_2_–MWCNTs electrode exhibited the largest peak current and the smallest ΔEp. The results indicate that Ti_3_C_2_ and MWCNTs effectively increased the electron transfer efficiency of the modified electrode. In addition, the reaction kinetics of the 3D rGO–Ti_3_C_2_–MWCNTs electrode was investigated. The peak current gradually enhanced with an increasing scan rate (Figure 2B). Both oxidation and reduction peak currents against the square root of the scan rate showed a good linear relationship (inset of Figure 2B), indicating that the catalytic process was a diffusion-controlled process [26].

The effective surface area of 3D rGO, rGO–Ti_3_C_2_, rGO–MWCNTs, and rGO–Ti_3_C_2_-MWCNTs was calculated using the Randles–Sevcik equation as follows:

I*_p_* = (2.69 × 10^5^) A*_eff_ n*^3/2^ D_0_^1/2^ C_0_ v^1/2^

where I*p* represents the peak current; A*_eff_* is the effective surface area; n is the number of electrons transferred between the electrode and electrolyte, which is equal to 1; D_0_ is the diffusion coefficient in the solution (D_0_ = 0.673 × 10^−5^ cm^2^ s^−1^); v is the scan rate; and C_0_ is the concentration of the analyte (C_0_ = 5 × 10^−6^ mol cm^−3^). Based on this equation, the electroactive surface area of 3D rGO, rGO–Ti_3_C_2_, rGO–MWCNTs, and rGO–Ti_3_C_2_–MWCNTs electrodes were calculated as 0.0096 cm^2^, 0.0143 cm^2^, 0.01205 cm^2^, and 0.0214 cm^2^, respectively. The results suggested that the simultaneous incorporation of Ti_3_C_2_ and MWCNTs can significantly enhance the specific surface area of the modified electrode.

### 3.3. Electrocatalytic Reduction of H_2_O_2_ by the 3D rGO–Ti_3_C_2_–MWCNTs Electrode

**Figure 3:**
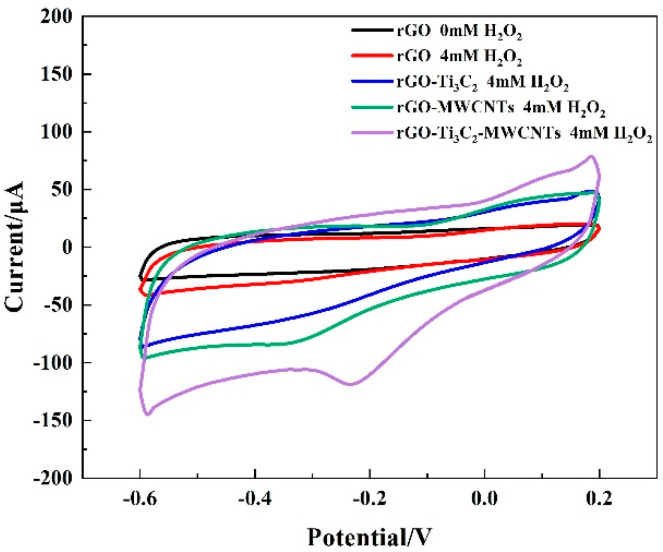
CV of 3D rGO, rGO–Ti_3_C_2_, rGO–MWCNTs, and rGO–Ti_3_C_2_–MWCNTs electrodes in deoxygenated 0.01 M PBS in the presence and absence of 4 mM H_2_O_2_.

The electrocatalytic properties of the modified electrode toward H_2_O_2_ reduction were first investigated by CVs. With the gradual increase in H_2_O_2_, the cathodic currents of 3D rGO, rGO–Ti_3_C_2_, rGO–MWCNTs, and rGO–Ti_3_C_2_–MWCNTs electrodes showed an obvious increase, indicating that H_2_O_2_ was reduced on the modified electrode (Figure S2). The introduction of Ti_3_C_2_ and MWCNTs results in anodic steps for rGO–Ti_3_C_2_, rGO–MWCNTs, and rGO–Ti_3_C_2_–MWCNTs electrodes (Figure S4). No significant cathode reduction peaks were observed on 3D rGO and rGO–Ti_3_C_2_ electrodes. Significant H_2_O_2_ reduction peaks at −0.37 V and −0.25 V could be observed on 3D rGO–MWCNTs and rGO–Ti_3_C_2_–MWCNTs, respectively (Figure 3). Increased reduction current and reduced reduction potential indicated that the 3D rGO–Ti_3_C_2_–MWCNTs electrode could provide an ideal performance for reduction-based H_2_O_2_ detection.

### 3.4. Amperometric Performance of the 3D rGO–Ti_3_C_2_–MWCNTs Electrode

**Figure 4:**
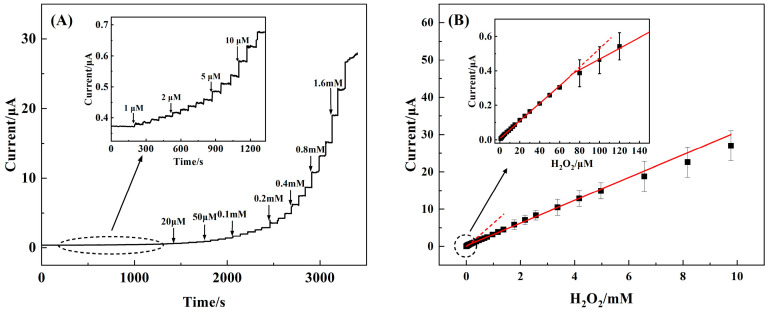
(**A**) Amperometric response of the 3D rGO–Ti_3_C_2_–MWCNTs electrode upon adding H_2_O_2_ in deoxygenated 0.01 M PBS at a constant potential of −0.25 V under continuous stirring (inset: expand view at 1, 2, 5, and 10 µM H_2_O_2_). (**B**) Calibration curve of the 3D rGO–Ti_3_C_2_–MWCNTs electrode current vs. H_2_O_2_ concentration (inset: calibration curve showing H_2_O_2_ concentration from 1–60 µM).

The amperometric response of the 3D rGO–Ti_3_C_2_–MWCNTs electrode was recorded (Figure 4A). Three to five replicate tests were performed at each experimental point to obtain error bars. The current response of the 3D rGO–Ti_3_C_2_–MWCNTs electrode showed good linearity in the H_2_O_2_ concentration ranges of 1 μM−60 μM (R^2^ = 0.998) and 60 μM−9.77 mM (R^2^ = 0.992), with sensitivities of 235.2 µA mM^−1^ cm^−2^ and 103.8 µA mM^−1^cm^−2^, respectively, and a limit of detection of 0.3 µM (S/N = 3) (Figure 4B). Further, a comparison of the 3D rGO–Ti_3_C_2_–MWCNTs electrode with other H_2_O_2_ sensors is summarized in Table 1. Compared with the other literature, the 3D rGO–Ti_3_C_2_–MWCNTs electrode had the advantage of a low detection limit.

### 3.5. Repeatability, Stability, and Selectivity of the 3D rGO–Ti_3_C_2_–MWCNTs Electrode

**Figure 5:**
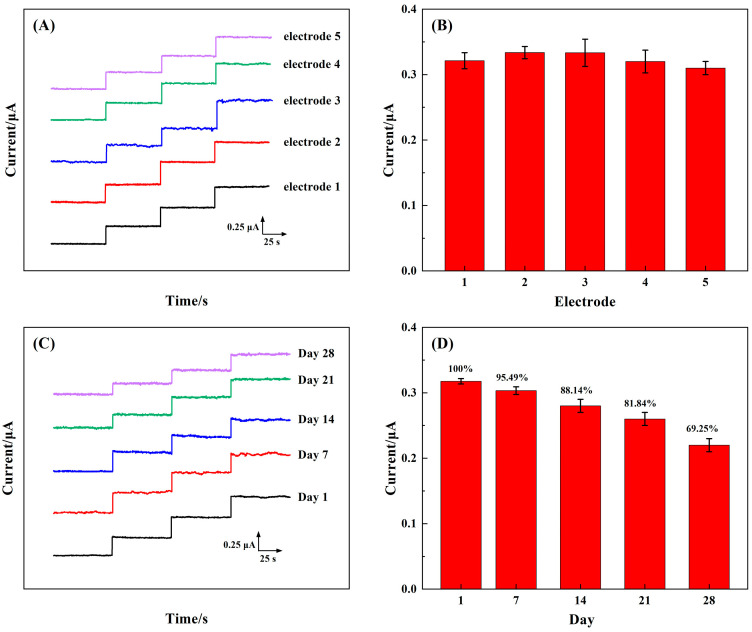
(**A**) Reproducibility study of the 3D rGO–Ti_3_C_2_–MWCNTs electrode at a constant potential of −0.25 V with continuous addition of 100 μM H_2_O_2_ in deoxygenated 0.01 M PBS. (**B**) Reproducibility evaluation of five 3D rGO–Ti_3_C_2_–MWCNTs electrodes. (**C**) Long-term (4 weeks) stability of the 3D rGO–Ti_3_C_2_–MWCNTs electrode at a constant potential of −0.25 V with continuous addition of 100 μM H_2_O_2_ in deoxygenated 0.01 M PBS. (**D**) Stability assessment of the 3D rGO–Ti_3_C_2_–MWCNTs electrode for 4 weeks.

The reproducibility of the 3D rGO–Ti_3_C_2_–MWCNTs electrode was also investigated. The responses were very similar for all five electrodes (Figure 5A). The electrode-to-electrode reproducibility was characterized by the low relative standard deviation (RSD) of 3.08% (*n* = 5) in response to 100 μM H_2_O_2_. The RSD of 1.61% (*n* = 3) for 100 μM H_2_O_2_ using the same electrode demonstrated good intra-electrode reproducibility. The long-term stability of the 3D rGO–Ti_3_C_2_–MWCNTs electrode was demonstrated by periodically recording the amperometric response to H_2_O_2_ (Figure 5C). The reduction current to H_2_O_2_ was maintained at 95.49% after 7 days and 88.14% after 14 days (Figure 5D). This result indicated that 3D rGO–Ti_3_C_2_–MWCNTs had excellent stability.

**Figure 6:**
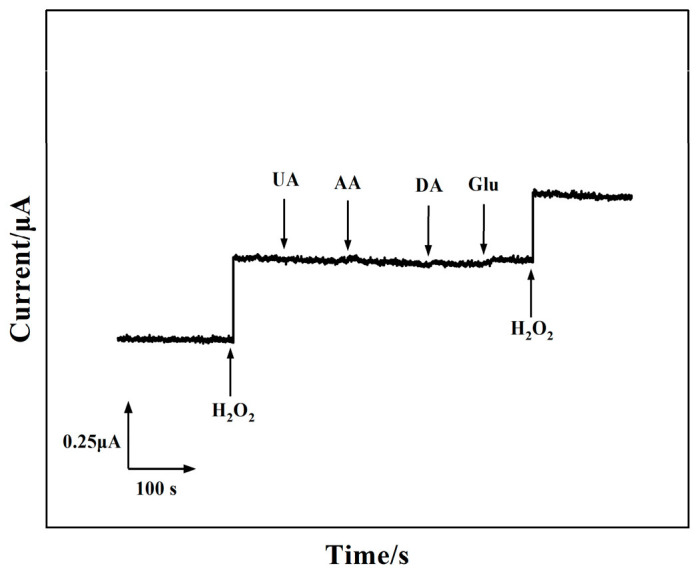
Selectivity of the 3D rGO–Ti_3_C_2_–MWCNTs electrode to 100 µM H_2_O_2_, 200 µM interfering species (UA, AA, DA, Glu), and 100 µM H_2_O_2_ in deoxygenated 0.01 M PBS at a constant potential of −0.25 V.

Immunity to interference was a very important factor for in vivo detection. Several common interferences, such as UA, AA, DA, and glucose (Glu), were added during the detection of H_2_O_2_. The current response of the 3D rGO–Ti_3_C_2_–MWCNTs electrode was recorded upon successive additions of 100 µM H_2_O_2_, 200 µM interfering species, and then 100 µM H_2_O_2_ again in the deoxygenated 0.01 M PBS solution. The 3D rGO–Ti_3_C_2_–MWCNTs electrode produced almost no current response when the interfering substance was added, while the current response to H_2_O_2_ was unaffected (Figure 6). The results indicated that the 3D rGO–Ti_3_C_2_–MWCNTs electrode had high interference resistance.

### 3.6. Ex Vivo Experimental Analysis

The 3D rGO–Ti_3_C_2_–MWCNTs electrode was used for real-time in vitro detection of H_2_O_2_ in cells. MCF−7 and 4T1 cells were used as model cells to test H_2_O_2_ release upon fMLP stimulation in real time [52,53,54]. Normal cells can produce H_2_O_2_ at concentrations of up to 0.05~0.7 μM in a physiological environment. In breast cancer, H_2_O_2_ production is several times higher than in normal cells [46,55]. The deoxygenated 0.01 M PBS containing 4T1 or MCF−7 cells was supplemented with 50 μL of fMLP (1 mg mL^−1^). The amperometric current of 4T1 cells exhibited a significant increase of 20.5 nA, corresponding to the production of 2.4 μM H_2_O_2_ calculated by the calibration curve in Figure 4B. Similarly, MCF−7 cells demonstrated a notable rise in amperometric current by 25.4 nA, indicating the generation of 3.4 μM H_2_O_2_. The current significantly decreased with catalase injection. As controls, the currents were also recorded following the injection of fMLP-free PBS and the injection of fMLP into PBS without cells. This phenomenon suggested that the reduction current increase was caused by H_2_O_2_ released from cancer cells. The same amount of fMLP was added to EPH4−EV cells (normal cells). As shown in Figure S6, H_2_O_2_ produced by normal cells was much lower than that produced by cancer cells. These results indicated that 3D rGO–Ti_3_C_2_–MWCNTs electrodes can effectively discriminate between normal cells and tumor cells in order to detect cancer.

**Figure 7:**
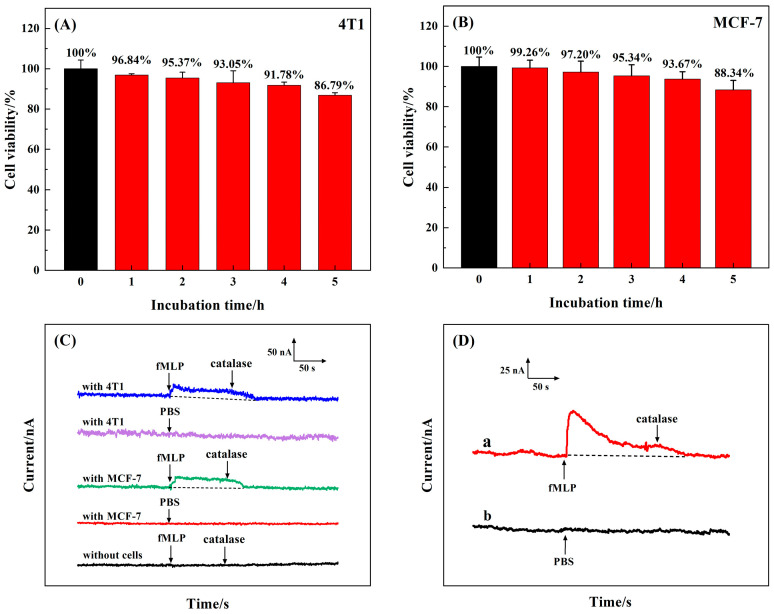
Quantitative cell viability results by CCK−8 assay for (**A**) 4T1 and (**B**) MCF−7 cells incubated with the 3D rGO–Ti_3_C_2_–MWCNTs electrode for 0, 1, 2, 3, 4, and 5 h. (**C**) Reduction current response of the 3D rGO–Ti_3_C_2_–MWCNTs electrode with the addition of fMLP, catalase, and fMLP-free PBS to deoxygenated 0.01 M PBS solution containing 7.0 × 10^6^ 4T1 cells, 5.0 × 10^6^ MCF−7 cells, and no cells. (**D**) Amperometric current response of the 3D rGO–Ti_3_C_2_–MWCNTs electrode after separate injections of fMLP, catalase, and fMLP-free PBS into breast cancer tissue immersed in the deoxygenated 0.01 M PBS solution.

Subsequently, the real-time monitoring of H_2_O_2_ in mouse cancer tissue was successfully achieved through the utilization of the 3D rGO–Ti_3_C_2_–MWCNTs electrode. The modified electrode was inserted inside the breast cancer tissue, and a significant current increase was detected after fMLP injection. Then, the reduction current response gradually decreased with catalase injection (Figure 7D). The amperometric current of mouse cancer tissue exhibited a significant increase of 19.8 ± 3.2 nA, corresponding to the production of 2.3 μM H_2_O_2_ calculated by the calibration curve in Figure 4B. As controls, the injection of fMLP-free PBS into tissue cultures did not result in any observed change. The findings indicated that breast cancer tissue produced H_2_O_2_ upon fMLP stimulation and subsequently eliminated it through the addition of catalase. Therefore, the 3D rGO–Ti_3_C_2_−MWCNT electrode provided an effective idea for the ex vivo detection of tumor biomarkers with practical applications.

## 4. Conclusions

A 3D rGO–Ti_3_C_2_–MWCNTs electrode was fabricated through hydrothermal reduction and utilized for the detection of H_2_O_2_. The modified electrode was successfully utilized for real-time monitoring of the release of H_2_O_2_ from cancer cells and tissue. The experimental data demonstrated a significant enhancement in the electrocatalytic activity of the 3D rGO–Ti_3_C_2_–MWCNTs electrode to H_2_O_2_ following the simultaneous incorporation of Ti_3_C_2_ and MWCNTs into rGO. In addition, the rGO–Ti_3_C_2_–MWCNTs electrode exhibited excellent selectivity, stability, and reproducibility in the detection of H_2_O_2_. The ex vivo detection of H_2_O_2_ in cancer tissue was conducted, which demonstrated exceptional current response. The present study introduces a novel approach to histological examination of cancerous tissue post-biopsy, thereby providing the medical community with innovative insights into early cancer detection.

## Figures and Tables

**Table 1:** Table of comparison of the 3D rGO–Ti_3_C_2_–MWCNTs electrode with other H_2_O_2_ sensors.

Electrode	Method	Linear Range (mM)	LOD (µM)	Sensitivity (µA mM^−1^ cm^−2^)	Refs.
CoFe_2_O_4_ HS/GCE	Amperometry	0.01–1.2	2.5	715	[44]
rGO/Ag/PdNPs/GCE	Amperometry	0.005–14.65	1.1	342	[45]
PtPd/MWCNT/GCE	Amperometry	0.0025–0.125	1.2	414.8	[22]
MoS_2_–Au–Ag/GCE	Amperometry	0.05–20	7.19	405.2	[46]
Nafion/Gr–CCS–AgNPs/GCE	Amperometry	0.02–5.02	2.49	-	[47]
Pd-PANi/CMs	Amperometry	0.002–10	0.7	234	[48]
Catalase/MoS_2_–Au/chitosan/GCE	Amperometry	0.0005–0.2	0.1	187.4	[49]
Fer/rGO–Pt	Amperometry	0.0004–0.01 0.0075–4.27 4.89–10.77	0.228	340	[50]
MX/CS/PB/GCE	Amperometry	0.0005–0.667	0.004	-	[51]
rGO–Ti_3_C_2_–MWCNTs	Amperometry	0.001–9.77	0.3	235.2	This work

GCE: glassy carbon electrode; CoFe_2_O_4_ HS: CoFe_2_O_4_ hollow spheres; PdNP: palladium nanoparticles; Gr–CCS–AgNPs: graphene-colloidal carbon sphere composite decorated with silver nanoparticles; PANi: polyaniline; CMs: colloidal micro-catalyst; Fer: ferumoxytol; MX: Ti_3_C_2_T_x_; CS: PB: Chitosan; PB: Prussian blue.

## Data Availability

Data are contained within the article.

## Supplementary Materials

The following supporting information can be downloaded at: https://www.mdpi.com/article/10.3390/bios14060261/s1, Figure S1: XRD pattern (A) and XPS spectra (B) of 3D rGO, rGO–Ti_3_C_2_, rGO–MWCNTs, and rGO–Ti_3_C_2_–MWCNTs; Figure S2: CV of (A) 3D rGO, (B) 3D rGO–Ti_3_C_2_, (C) 3D rGO–MWCNTs, and (D) 3D rGO–Ti_3_C_2_–MWCNTs electrodes at different H_2_O_2_ concentrations (0–10 mM) in deoxygenated 0.01 M PBS. Scan rate: 100 mV s^−1^. Potential range: −0.6–0.2 V; Figure S3: Calibration curves of 3D rGO, rGO–Ti_3_C_2_, rGO–MWCNTs, and rGO–Ti_3_C_2_–MWCNTs electrodes currents vs. H_2_O_2_ concentration; Figure S4: CV of 3D rGO, rGO–Ti_3_C_2_, rGO–MWCNTs, and rGO–Ti_3_C_2_–MWCNTs electrodes in deoxygenated 0.01 M PBS in the presence and absence of 4 mM H_2_O_2_; Figure S5: (A) Current response of 3D rGO–Ti_3_C_2_–MWCNTs electrode to 100 μM H_2_O_2_ in deoxygenated 0.01M PBS (pH 7.0) at different potentials. (B) Influence of applied potential on amperometric response of the biosensor. (C) Change in amperometric current of the electrode against 100 μM H_2_O_2_ at different pH values of PBS. (D) Dependence of the current response of 3D rGO–Ti_3_C_2_–MWCNTs electrode to 100 μM H_2_O_2_ on the pH of buffer solutions at an applied potential of −0.25 V vs. Ag/AgCl; Figure S6: Reduction current response of the 3D rGO–Ti_3_C_2_–MWCNTs electrode with addition of fMLP to deoxygenated 0.01 M PBS solution containing (A) 4T1, (B) MCF−7 and (C) EPH4−EV cells. (D) EPH4−EV, 4T1 and MCF−7 cells produced hydrogen peroxide concentrations by addition of the same amount of fMLP. References [11,56,57,58,59] are cited in the supplementary materials.
