# Supplementary material for: Highly Sensitive Detection of Hydrogen Peroxide in Cancer Tissue Based on 3D Reduced Graphene Oxide–MXene–Multi-Walled Carbon Nanotubes Electrode
Source: Biosensors (Basel). 2024 May 21;14(6):261. doi: 10.3390/bios14060261 (PMC11201644; doi:10.3390/bios14060261)
Supplement: Supplementary file 1 [file biosensors-14-00261-s001.zip › biosensors-2980673-supplementary.pdf]

biosensors

MDPI

Article

Highly Sensitive Detection of Hydrogen Peroxide in Cancer Tissue Based on 3D Reduced Graphene Oxide–MXene–Multi-Walled Carbon Nanotubes Electrode

Shuai-Qun Yu, Pan Li, Hao-Jie Li, Ling-Jun Shang, Rui Guo, Xu-Ming Sun * and Qiong-Qiong Ren *

School of Medical Engineering, Xinxiang Medical University, Xinxiang, Henan 453003, China; m15225998391@163.com (S.-Q.Y.); kxx0806@163.com (P.L.); 18436264383@163.com (H.-J.L.); slj1216002129@163.com (L.-J.S.); pyguorui@sina.com (R.G.)

* Correspondence: sunxuming@xxmu.edu.cn (X.M.S.); 151036@xxmu.edu.cn (Q-Q.R.); Tel.: +86-373-3831929 (Q.-Q.R.)

1. XRD and XPS characterization

Structures of 3D rGO, rGO–Ti_3C_2, rGO–MWCNTs and rGO–Ti_3C_2–MWCNTs were studied by X–ray diffraction (XRD). The characteristic peaks of graphite structure appear at about 25° and 43° for rGO and MWCNTs, which correspond to the diffraction peaks of carbon atoms at (002) and (100), respectively [1, 2]. And characteristic peaks at 26°, 38°, 48°, 55°, and 63° correspond to the diffraction peaks of Ti_3C_2 at (004), (103), (107), (109), and (110) (Figure S1A) [3].

XPS measurements were carried out to study the composition of 3D rGO, rGO–Ti_3C_2, rGO–MWCNTs and rGO–Ti_3C_2–MWCNTs. The results showed that 3D rGO–Ti_3C_2–MWCNTs samples contained major elements such as C, O, and Ti (Figure S1B). Thus, the success of interaction between rGO, Ti_3C_2 and MWCNTs was proved to form 3D rGO–Ti_3C_2–MWCNTs nanocomposites.

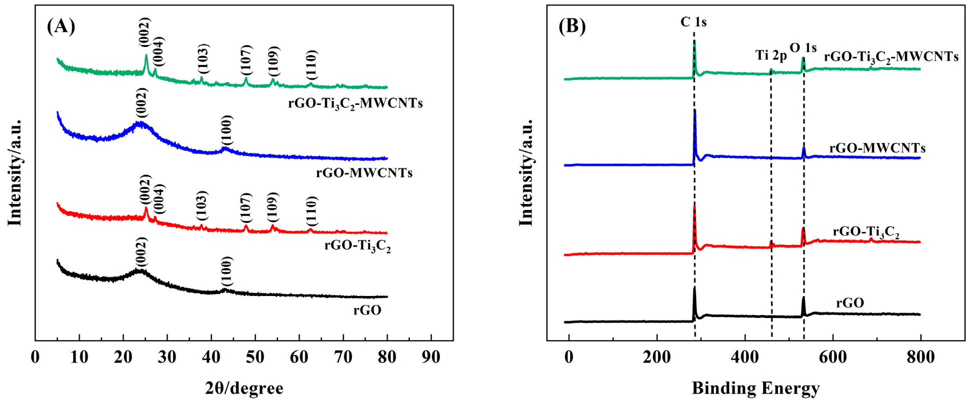

Figure S1. XRD pattern (A) and XPS spectra (B) of 3D rGO, rGO–Ti_3C_2, rGO–MWCNTs, and rGO–Ti_3C_2–MWCNTs.

2. Electrocatalytic reduction of H_2O_2

In order to study the electrocatalytic reduction of H_2O_2, CV scans of 3D rGO, rGO–Ti_3C_2, rGO–MWCNTs and rGO–Ti_3C_2–MWCNTs electrodes were carried out at different H_2O_2 concentrations in deoxygenated 0.01 M PBS solution (Figure S2). The current response values exhibited a linear correlation with the H_2O_2 concentration for all four electrodes at a potential of −0.25 V (Figure S3). Among them, 3D rGO–Ti_3C_2–MWCNTs electrode demonstrated the highest current response to H_2O_2. The results indicated that 3D rGO–Ti_3C_2–MWCNTs electrode had more efficient electrocatalytic efficiency for H_2O_2.

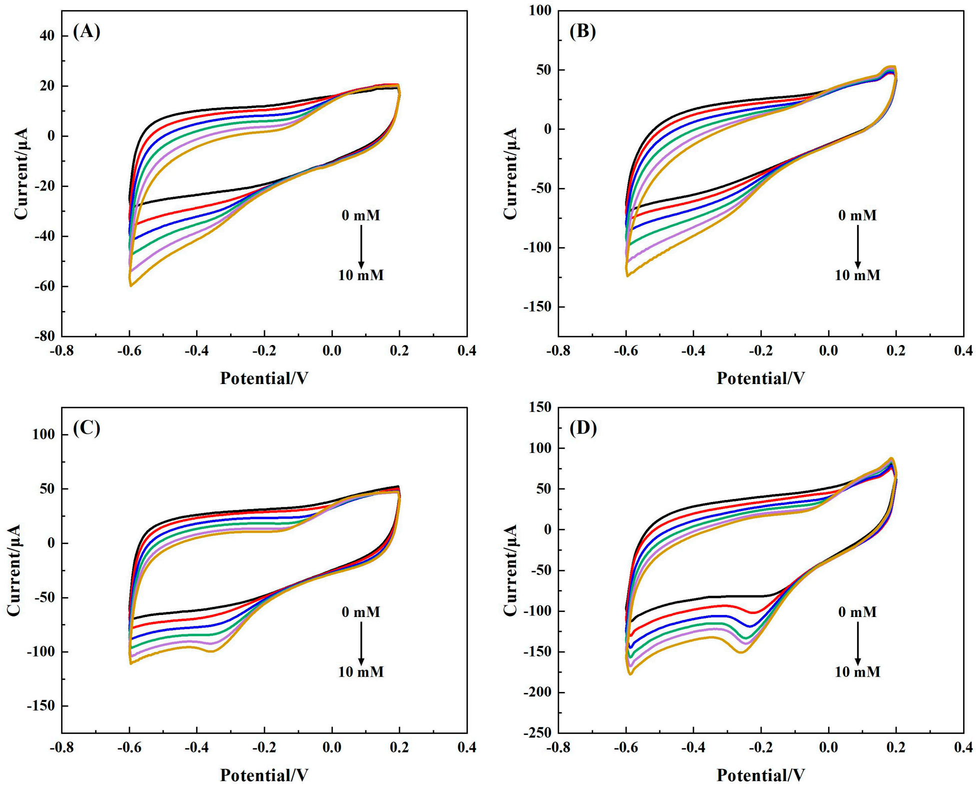

Figure S2. CV of (A) 3D rGO, (B) 3D rGO−Ti_3C_2, (C) 3D rGO−MWCNTs, and (D) 3D rGO−Ti_3C_2−MWCNTs electrodes at different H_2O_2 concentrations (0−10 mM) in deoxygenated 0.01 M PBS. Scan rate: 100 mV s^{-1}. Potential range: −0.6−0.2 V.

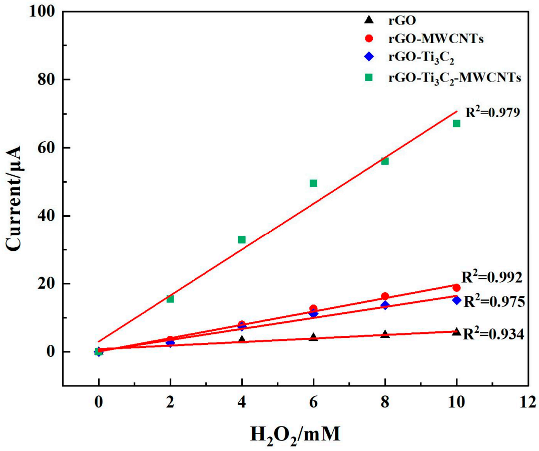

Figure S3. Calibration curves of 3D rGO, rGO−Ti_3C_2, rGO−MWCNTs, and rGO−Ti_3C_2−MWCNTs electrodes currents vs. H_2O_2 concentration.

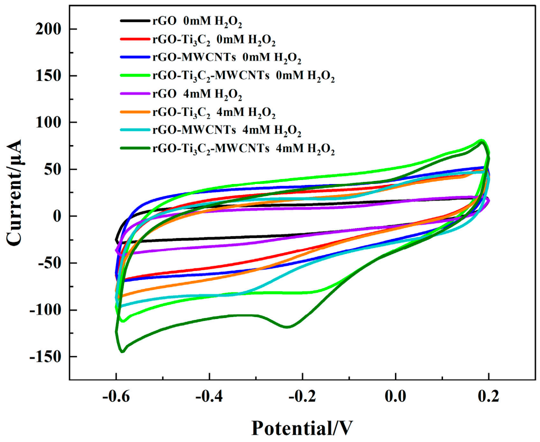

Figure S4. CV of 3D rGO, rGO−Ti_3C_2, rGO−MWCNTs, and rGO−Ti_3C_2−MWCNTs electrodes in deoxygenated 0.01 M PBS in the presence and absence of 4 mM H_2O_2.

3. Optimization of test conditions for 3D rGO−Ti_3C_2−MWCNTs electrode

The dependence of 3D rGO−Ti_3C_2−MWCNTs electrode response on applied potential is shown in Figure S5A. The reduction current tends to maximum on −0.25 V (Figure S5B), thus this value is selected as the working potential. The influence of pH on the response of 3D rGO−Ti_3C_2−MWCNTs electrode was investigated to optimize the reaction conditions. The change of amperometric current between the pH level of 5.0–9.0 at a constant H_2O_2 concentration (100 μM H_2O_2) is shown in Figure S5C. The maximum response was obtained at pH 7.0 (Figure S5D). Thus, the optimized pH level of 7.0 was used through further experiments [4, 5].

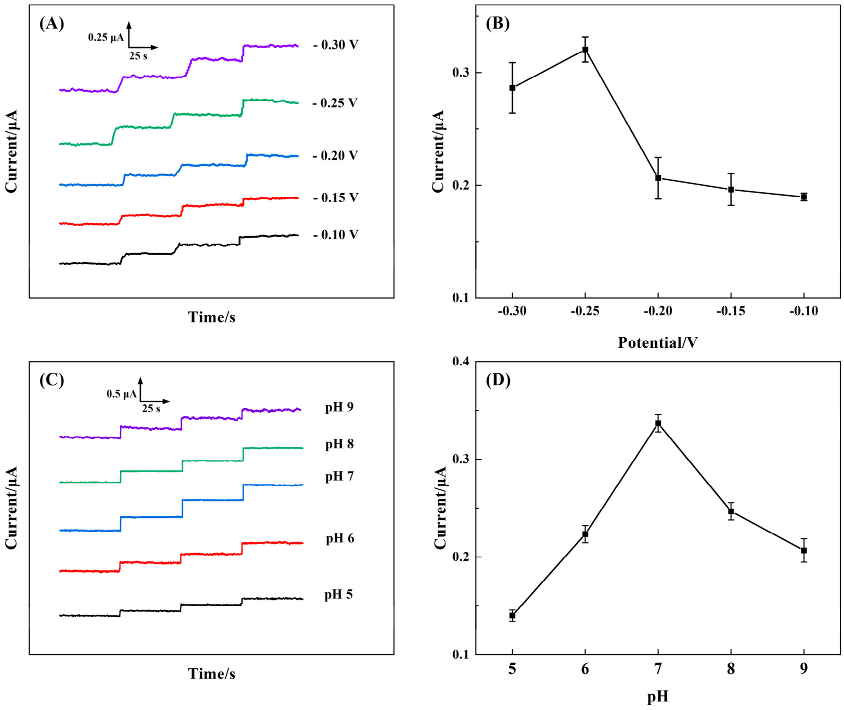

Figure S5. (A) Current response of 3D rGO−Ti_3C_2−MWCNTs electrode to 100 μM H_2O_2 in deoxygenated 0.01M PBS (pH 7.0) at different potentials. (B) Influence of applied potential on amperometric response of the biosensor. (C) Change in amperometric current of the electrode against 100 μM H_2O_2 at different pH values of PBS. (D) Dependence of the current response of 3D rGO−Ti_3C_2−MWCNTs

electrode to 100 μM H_2O_2 on the pH of buffer solutions at an applied potential of −0.25 V vs. Ag/AgCl.

4. Ex vivo experimental analysis

The same amount of fMLP is added to EPH4−EV cells (normal cell). 4T1, MCF−7 and EPH4−EV cell lines produced a current response of 20.5±3.4 nA, 25.4±2.1 nA, and 7.3±1.5nA respectively. Based on the calibration curves in Fig. 4B, these values correspond to the production of 2.4 μM H_2O_2, 3.4 μM H_2O_2, and 0.9 μM H_2O_2, respectively. As shown in Figure S6, H_2O_2 produced by normal cells was much lower than that produced by cancer cells. The results indicate 3D rGO−Ti_3C_2−MWCNTs electrodes can effectively discriminate between normal cells and tumor cells in order to detect cancer.

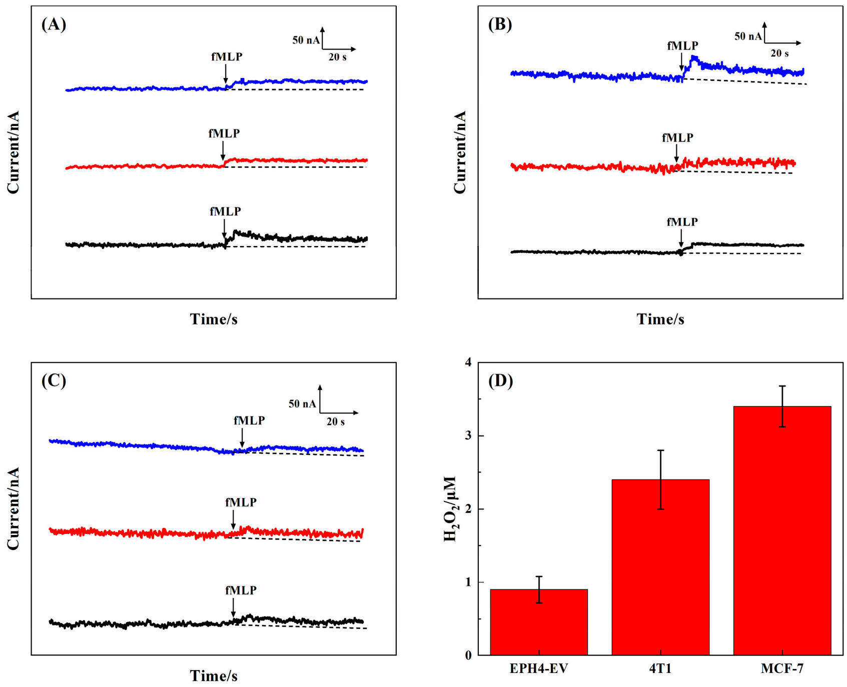

Figure S6. Reduction current response of the 3D rGO−Ti_3C_2−MWCNTs electrode with addition of fMLP to deoxygenated 0.01 M PBS solution containing (A) 4T1, (B) MCF−7 and (C) EPH4−EV cells. (D) EPH4−EV, 4T1 and MCF−7 cells produced hydrogen peroxide concentrations by addition of the same amount of fMLP.

References

1. Su, Y.; Song, H.; Yi, L. Recent advances in chemiluminescence for reactive oxygen species sensing and imaging analysis. *Microchemical Journal: Devoted to the Application of Microtechniques in all Branches of Science.* **2019**, *146,* 83-97. [CrossRef]
2. Chen, S.; Shi, M.; Xu, Q.; Xu, J.; Duan, X.; Gao, Y.; Lu, L.; Gao, F.; Wang, X.; Yu, Y. Ti(3)C(2)T(x)MXene/nitrogen-doped reduced graphene oxide composite: a high-performance electrochemical sensing platform for adrenaline detection. *Nanotechnology.* **2021**, *32,* 26. [CrossRef].
3. Wang, L.; Xiao; F.; Xiong, Q.; Duan, H. 2D nanomaterials based electrochemical biosensors for cancer diagnosis. *Biosensors Bioelectronics.* **2017**, *89 (1),* 136-151. [CrossRef]
4. Tang, C.; Zhang, J.-X.; Chen, D.-N.; He, J.-W.; Wang, A.-J.; Feng, J.-J. Ultrasensitive label-free electrochemical immunosensor of NT-proBNP biomarker based on branched AuPd nanocrystals/N-doped honeycombed porous

carbon. *Bioelectrochemistry.* **2022**, *148* 108225. [CrossRef]

5. Tian, C.; Zhang, S.; Zhuang, X.; Wang, H.; Chen, D.; Luan, F.; He, T.; Qiu, Y. Preparation of gold nanoparticles supported on graphene oxide with flagella as the template for nonenzymatic hydrogen peroxide sensing. *Analytical Bioanalytical Chemistry.* **2018**, *410* 5915-5921. [CrossRef]
